# Incidence of local complications following implementation of alcoholic chlorhexidine for peripheral venous catheter site disinfection

**DOI:** 10.1017/ash.2025.10170

**Published:** 2025-10-14

**Authors:** Ventsislava Berg, Martin Nufer, Michaela Gligor, Christina Orasch, Rami Sommerstein

**Affiliations:** 1 Faculty of Health Sciences and Medicine, University of Lucerne, Lucerne, Switzerland; 2 Rehaklinik Zentralschweiz, Klinik Adelheid, Zug, Unterägeri, Switzerland; 3 Clinic St. Anna, Lucerne, Switzerland; 4 Medsyn SA, Lucerne, Switzerland; 5 Department of Infectious Diseases, Bern University Hospital, University of Bern, Bern, Switzerland

## Abstract

A previous controlled study showed advantages of 2% Chlorhexidine Gluconate-Alcohol (CHG) over Povidone-Iodine-Alcohol in preventing infections after peripheral venous catheter placement. We applied these findings in a real-world before/after healthcare intervention and found that introduction of CHG disinfection was not associated with a major change of incidence in local skin complications.

## Introduction

Peripheral venous catheters (PVCs) are the most commonly used invasive devices in hospital settings, with up to 70% of hospitalized patients receiving a PVC^1^ and approximately 2 billion PVCs sold worldwide annually.^2^ While generally considered low-risk devices, PVCs can occasionally lead to serious adverse events, including mechanical, vascular, and infectious complications.

Research on strategies to reduce PVC-associated local complications has identified several risks: Key factors influencing complication rates include hand hygiene compliance, vein selection, puncture technique, skin disinfection, and regular inspection of the insertion site.^3,4^ Among available skin disinfectants, chlorhexidine stands out as a broad-spectrum antibacterial agent effective against both Gram-positive and Gram-negative bacteria, with additional activity against yeasts and dermatophytes.^5^

Previous studies have demonstrated that 2% chlorhexidine gluconate in 70% alcohol (CHG) outperforms other alcohol-based disinfectants in reducing infection-related complications.^6^ Our study aimed to evaluate whether implementing CHG as a routine disinfectant in a real-world setting would impact local PVC site complication rates.

### Methods and findings

#### Study setting and intervention

Prior to 2023, the tertiary care hospital St. Anna (Lucerne, Switzerland) used isopropanol alcohol (IPA) as the standard skin disinfectant for PVC insertion. On February 1, 2023, following recommendations from the hospital hygiene department, all existing Kodan® forte bottles (containing isopropyl alcohol, propanol, and 2-phenylphenol) were replaced with Softasept® chlorhexidine 2% solution. This transition was preceded by staff communication, updates to standard operating procedures, and appropriate training.

#### Monitoring and documentation

The nursing staff maintained consistent, prospective monitoring practices throughout both periods, documenting local findings as long as the PVC was in place (suppuration, redness, vein hardening) three times daily in the electronic health record system. Individual PVC episodes were recognized and defined through specific attributes such as insertion and removal data; side (left vs right); as well as anatomic position (eg, wrist, forearm, cubital fossa). Postremoval PVC surveillance was by definition not possible. Ward of insertion was recorded as well in the data set—all PVC inserted outside the institution were excluded from the study.

The hospital hygiene staff conducted periodic checks to ensure proper implementation and documentation. Check on the implementation of disinfectant distribution and use was not performed pre intervention, as CHG was not available for PVC site disinfection in the designated wards.

### Statistical analysis

We compared patient characteristics and outcome rates between the two periods using *χ* ^2^ tests for categorical data and Wilcoxon tests for continuous data. The primary outcome was a composite of local complications (redness, suppuration, and hardening). Sensitivity analysis included the composite redness and suppuration only, which can be viewed as a more reliable marker of possible PVC infection. We calculated the crude PVC outcome rate per 1 000 lay days, as well as the incidence rate ratio using Poisson regression, analyzing monthly complication rates before and after the intervention. Statistical significance was set at *P* < .05, using R version 4.1.2.

This quality assurance study was exempt from ethical review under Swiss law. According to the Swiss Human Research Act, quality assurance and quality control studies are classified as basic research activities protected under Article 20 of the Federal Constitution of the Swiss Confederation. Such studies do not require prior regulatory approval.

## Results

### Study population

After exclusion of 667 PVC inserted outside the institution, our analysis included 35,032 PVC episodes: 23 895 in the IPA group (January 2021–January 2023) and 11,137 in the CHG group (February 2023–January 2024). Both groups (Table 1) showed statistically not significantly different patient characteristics: Median PVC dwell time: 2 days in both groups. Sex distribution was 54.1% women (IPA) versus 54.8% (CHG) and the median age: 66 [IQR 51–77] years (IPA) versus 66 [IQR 51–77] years (CHG).

**Table 1.** Baseline characteristics and outcomes (composite and single events) per study period

	IPA-Group (January 2021–January 2023)	CHG-Group (February 2023–February 2024	*P*-value
PVC episodes	23 895	11 137	
Baseline characteristics			
Dwell time in days (median, [IQR])	2 [2, 3]	2 [2, 3]	<.001
Female sex	12 923 (54.1%)	6 106 (54.8%)	.197
Age in years (median, [IQR])	66 [51-77]	66 [51-77]	.139
Insertion (ward)			<.001
Emergency department	3 464 (14.5%)	1 314 (11.8%)	
Operationg room	12 502 (52.3%)	5 633 (50.6%)	
General wards	7 929 (33.2%)	4 190 (37.6%)	
Outcomes at the PVC local site			
Composite outcome (Suppuration, redness, and/or hardened)	317 (1.3%)	118 (1.1%)	.040
Composite outcome per 1000 PVC days	5.28 (317/60 021 days)	4.37 (118/27 033 days)	.085
Suppuration	4 (0.0%)	0 (0.0%)	.407
Redness	234 (1.0%)	94 (0.8%)	.244
Hardening	102 (0.4%)	29 (0.3%)	.022

### Primary outcome

The composite outcome rate of local complications for the IPA group was 317/23 895 (1.3%) PVC episodes and in the CHG group 118/11 137 (1.1%) PVC episodes (*P* = .040). Per PVC days, the crude rate for the composite outcome was 5.28/1 000 lay days and 4.37/1 000 for CHG (*P* = .085).

The detailed comparison of individual outcomes are listed in Table 1.

**Figure 1.**
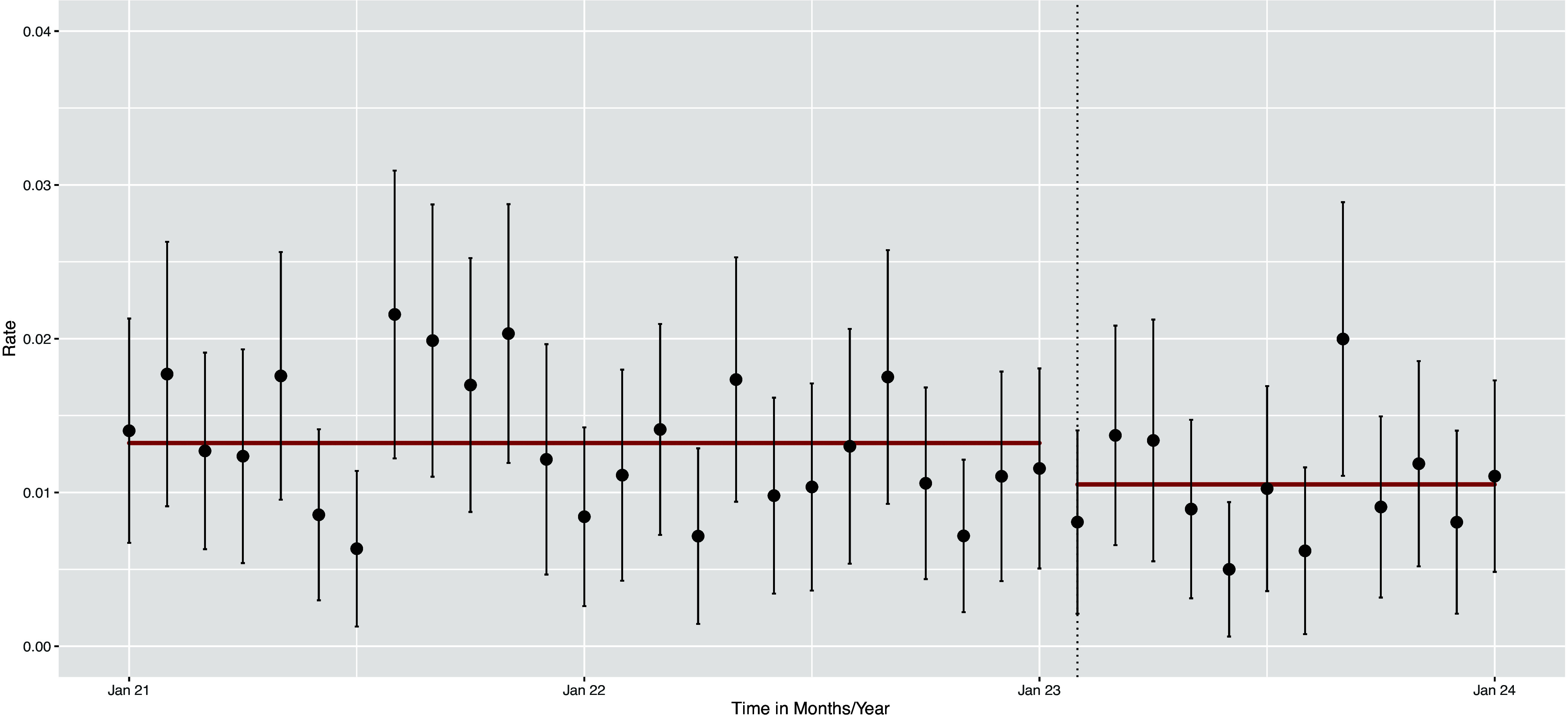
Incident rates per month with 95% CI and trendline per period (before/after intervention).

The incidence rate ratio after CHG implementation was 0.80 (95% CI, 0.64–0.98; *P* = .037), indicating a statistically significant reduction in complications (Figure 1).

In the sensitivity analysis (Supplementary Table and Supplementary Figure) focussing on the outcome suppuration and redness only, the IRR was 0.86 (95% CI 0.67–1.09, *P* = .21)

## Discussion

Our findings demonstrate that switching from IPA to CHG was associated with a marginally statistically significant reduction in local PVC complications, aligning with previous clinical trial data on CHG efficacy^3,8^. Taking into consideration the results of the sensitivity analysis, the statistically nonsignificant difference in the rate of outcome/1 000 PVC days and several limitations associated with this before/after study, our careful interpretation is, that the introduction of CHG disinfection was not associated with a major change of incidence in local skin complications.

While nursing adherence to insertion site monitoring was not directly measured, the consistent monthly PVC rates suggest stable compliance throughout the study period. To the best of our knowledge, there was no substantial change in other prevention methods throughout the study period, such as staff education on proper insertion, hand hygiene compliance, and sterile dressing options. Accordingly, we do not expect significant confounding due to other prevention methods introduced throughout the study period.

Study limitations include: i) Confounding by the study design (active change to a potentially better disinfectant with more allergies may lead to under/overreporting of outcomes); ii) inability to link microbiological results and mortality data due to separate database systems: these markers could have represented stronger biological correlation of poor or limited skin antisepsis; iii) lack of knowledge of concomitant antimicrobial therapy that may occult PVC outcomes (even though we do not believe, this is an effect modifier); iv) the role of the COVID pandemic and other time-dependent changes in infection-prevention practice that may have confounded results; and v) potential limitations in generalizability to facilities with different service ranges or microbial spectra.

## Conclusion

The implementation of CHG as the standard skin disinfectant was not associated with a major change of incidence in local skin complications in our before/after healthcare intervention. We rather consider the result or our study as hypothesis-generating than practice changing.

## Supporting information

10.1017/ash.2025.10170.sm001 Berg et al. supplementary material Berg et al. supplementary material

## Supplementary material

The supplementary material for this article can be found at https://doi.org/10.1017/ash.2025.10170.
