# Supplementary material for: Incidence of local complications following implementation of alcoholic chlorhexidine for peripheral venous catheter site disinfection
Source: Antimicrob Steward Healthc Epidemiol. 2025 Oct 14;5(1):e260. doi: 10.1017/ash.2025.10170 (PMC12538344; doi:10.1017/ash.2025.10170)

# Supplementary Materials

*Content***:**

Supplementary Table 1: Baseline characteristics and outcomes (for the sensitivity analysis with the composite outcome (redness and/or suppuration) per study period

Supplementary Figure 1: Incident rates per month with 95% CI and trendline per period (before/after intervention) for the sensitivity analysis with the composite outcome redness and/or suppuration

# Supplementary Table 1

Baseline characteristics and outcomes (for the sensitivity analysis with the composite outcome (redness and/or suppuration) per study period

|  | IPA-Group (January 2021-January 2023) | CHG-Group (February 2023-February 2024 | *P*-value |
| --- | --- | --- | --- |
| PVC episodes | 23 895 | 11 312 |  |
| Baseline characteristics | | | |
| Dwell time in days (median, [IQR]) | 2 [2, 3] | 2 [2, 3] | <0.001 |
| Female sex | 12 923 (54.1%) | 6 106 (54.8%) | 0.197 |
| Age in years (median, [IQR]) | 66 [51-77] | 66 [51-77] | 0.139 |
| Insertion (ward)  Emergency Dep.  Operationg room  General wards | 3 464 (14.5%)  12 502 (52.3%)  7 929 (33.2%) | 1 314 (11.8%)  5 633 (50.6%)  4 190 (37.6%) | <0.001 |
| Outcomes at the PVC local site | | | |
| Composite outcome (Suppuration, redness) | 235 (1.0%) | 94 (0.8%) | 0.230 |
| Suppuration | 4 (0.0%) | 0 (0.0%) | 0.407 |
| Redness | 234 (1.0%) | 94 (0.8%) | 0.244 |
| Composite outcome per 1000 PVC lay days | 3.91 (235/60 021 days) | 3.58 (94/27 033 days) | 0.360 |

Abbreviations:

PVC Peripheral Venous catheter

IPA Isopropanolalcohol

CHG 2% chlorhexedine gluconate in 70% alcohol

# Supplementary Figure 1

Incident rates per month with 95% CI and trendline per period (before/after intervention) for the sensitivity analysis with the composite outcome redness and/or suppuration


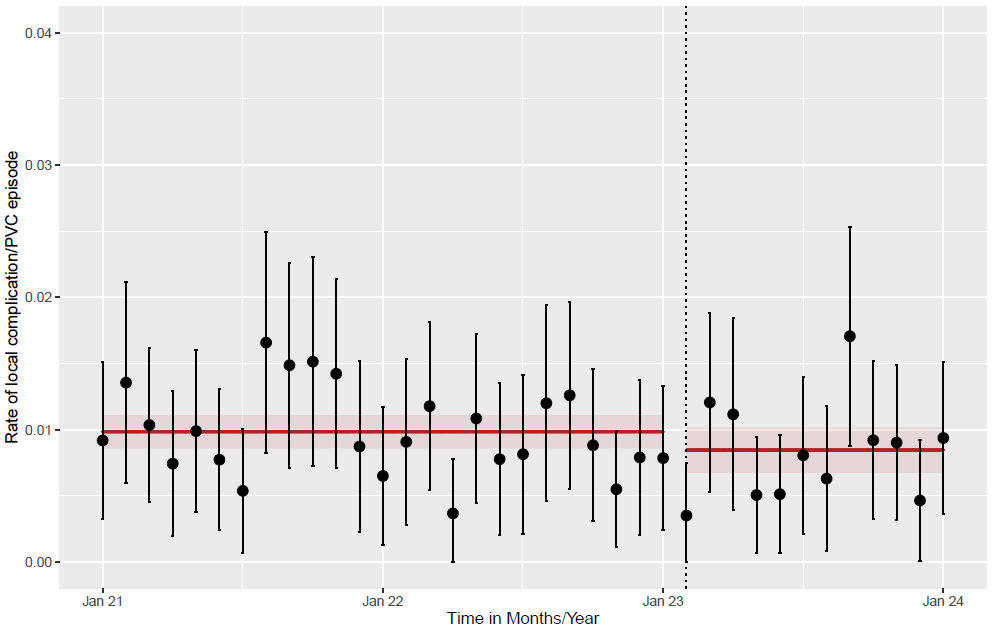

Supplement: Berg et al. supplementary material [file S2732494X25101708sup001.docx]
